# Human P2X7 receptor variants Gly150Arg and Arg276His polymorphisms have differential effects on risk association and cellular functions in pancreatic cancer

**DOI:** 10.1186/s12935-024-03339-9

**Published:** 2024-04-25

**Authors:** Lara Magni, Haoran Yu, Nynne M. Christensen, Mette H. Poulsen, Alexander Frueh, Ganga Deshar, Astrid Z. Johansen, Julia S. Johansen, Stephan A. Pless, Niklas R. Jørgensen, Ivana Novak

**Affiliations:** 1 Section for Cell Biology and Physiology, Department of Biology, University of Copenhagen, Universitetsparken 13, 2100 Copenhagen Ø, Denmark; 2 Department of Drug Design and Pharmacology, University of Copenhagen, Copenhagen, Denmark; 3 Department of Oncology, Copenhagen University Hospital - Herlev and Gentofte Hospital, Herlev, Denmark; 4 Department of Medicine, Copenhagen University Hospital – Herlev and Gentofte, Herlev, Denmark; 5 Department of Clinical Biochemistry, Copenhagen University Hospital Rigshospitalet, Copenhagen, Denmark; 6 Department of Clinical Medicine, Faculty of Health and Medical Sciences, University of Copenhagen, Copenhagen, Denmark

**Keywords:** Pancreatic ductal adenocarcinoma, SNP, P2X7R, Pancreatic stellate cells, Pancreatic cancer cells, IL-6

## Abstract

**Background:**

The purinergic P2X7 receptor (P2X7R) plays an important role in the crosstalk between pancreatic stellate cells (PSCs) and cancer cells, thus promoting progression of pancreatic ductal adenocarcinoma (PDAC). Single nucleotide polymorphisms (SNPs) in the P2X7R have been reported for several cancers, but have not been explored in PDAC.

**Materials and methods:**

Blood samples from PDAC patients and controls were genotyped for 11 non-synonymous SNPs in P2X7R and a risk analysis was performed. Relevant P2X7R-SNP GFP variants were expressed in PSCs and cancer cells and their function was assayed in the following tests. Responses in Ca^2+^ were studied with Fura-2 and dye uptake with YO-PRO-1. Cell migration was monitored by fluorescence microscopy. Released cytokines were measured with MSD assay.

**Results:**

Risk analysis showed that two SNPs 474G>A and 853G>A (rs28360447, rs7958316), that lead to the Gly150Arg and Arg276His variants, had a significant but opposite risk association with PDAC development, protecting against and predisposing to the disease, respectively. In vitro experiments performed on cancer cells and PSCs expressing the Gly150Arg variant showed reduced intracellular Ca^2+^ response, fluorescent dye uptake, and cell migration, while the Arg276His variant reduced dye uptake but displayed WT-like Ca^2+^ responses. As predicted, P2X7R was involved in cytokine release (IL-6, IL-1β, IL-8, TNF-α), but the P2X7R inhibitors displayed varied effects.

**Conclusion:**

In conclusion, we provide evidence for the P2X7R SNPs association with PDAC and propose that they could be considered as potential biomarkers.

**Supplementary Information:**

The online version contains supplementary material available at 10.1186/s12935-024-03339-9.

## Background

Pancreatic ductal adenocarcinoma (PDAC) has one of the highest mortality rates of all cancers [1–3] and is predicted to be the second leading cause of cancer death in 2030 [4]. The 5-year survival rate for patients with PDAC is only 13% for all stages [2]. Aggressiveness and late detection are the main challenges leading to therapeutic failure, with most of the patients presenting advanced or disseminated stages of the disease at the time of diagnosis [3]. PDAC is a solid, highly fibrotic, and hypo-vascular tumor, resistant to chemo/radiotherapy. The complex tumor microenvironment (TME) includes a variety of resident and recruited cells and non-cellular components, including, for example, cytokines and nucleotides/-sides. Due to the high metabolic activity of cancer cells, mechanical stress and necrosis, it is proposed that extracellular ATP concentrations can be relatively high in the TME [5]. Extracellular ATP activates many P2 purinergic receptors, and in particular one receptor, the P2X7R, is considered a potential treatment target in inflammation and some cancers [6]. P2X7R is expressed in several cell types in PDAC and can be targeted in mouse cancer models [7].

The P2X7R is a homo-trimeric ion channel, which consists of an extracellular, a transmembrane and an intracellular domain [8]. The extracellular domain of the receptor contains the ligand-binding site; the transmembrane domain constitutes the ion channel. The intracellular domain, which consists of a short N-terminus and a long C-terminus, is implicated in ERK and Ca^2+^ signaling, respectively, and in particular the C-terminus contains numerous motifs and interaction sites for downstream signaling [9–12]. The P2X7R is activated by extracellular ATP and stands out among other purinergic receptors for at least three reasons: first, it shows a lower apparent affinity for ATP compared with other subtypes; second, like some other subtypes, it can permeate molecules up to 900 Da and third, it contains a very long C-terminus [11, 13, 14].

The P2X7R gene, *P2RX7*, located on chromosome 12q24, is highly polymorphic. In addition to several splice variants [15], there are many single nucleotide polymorphisms (SNPs), some are in the coding region resulting in loss-of-function (LOF) or in gain-of-function (GOF) receptor phenotypes. For example, Gly150Arg, Glu186Lys and Ile568Asn (rs28360447, rs28360451, rs1653624) conferred complete loss of function when expressed in HEK293 cells, whereas His155Tyr and Ala348Thr (rs208294, rs1718119) caused a significant increase in both ATP-activated currents and pore formation [16]. Importantly, several of these P2X7R SNP variants are associated with various diseases including osteoporosis [17, 18], central nervous system disease [19], inflammation [20] and chronic pain [21]. In cancer, one of the most studied SNP variants is the LOF Glu496Ala (rs3751143) in B-chronic lymphocytic leukemia [22, 23]. Recently, several studies analyzed the role of P2X7R SNPs in other cancers, such as hepatocellular carcinoma, prostate cancer, and thyroid cancer [22, 24–26]. However, a similar study has not yet been performed in PDAC.

The P2X7R is expressed in many cell types, such as immune cells, epithelial cells, oligodendrocytes, and possibly neurons and astrocytes [11, 27]. In PDAC, P2X7R activation in pancreatic cancer cells promotes proliferation, migration in vitro as well as tumor growth and fibrosis in vivo [28, 29]. Recently, our studies showed that the receptor is expressed in pancreatic stellate cells (PSCs), the major fibrogenic cell type in TME, and promotes cell proliferation, collagen I secretion and IL-6 release, the latter stimulating cancer cells [30, 31]. However, overstimulation of the P2X7R causes cell death in both cancer cells and PSCs [28, 30, 31]. Moreover, and valid for cancers in general, P2X7Rs are expressed in immune cells and can support pro- and/or anti-tumorigenic effects, depending on the cancer stage and cancer type [6].

Our hypothesis is that P2X7R SNP variants show risk association in PDAC patients and specific genotypes could contribute to cancer occurrence and/or development. The first aim of this work was to carry out a case–control association study to explore SNPs occurrence in control and PDAC subjects. The second aim was to express relevant SNP variants in human pancreatic cancer and in stellate cells and evaluate their impact on cell functions. Here, we show that two SNPs variants (474G>A: Gly150Arg and 853G>A: Arg276His) have significant association with PDAC occurrence, the Gly150Arg variant being protective and the Arg276His variant promoting cancer, respectively. Furthermore, these two P2X7R SNPs showed different functional impacts when expressed in cancer cells and PSCs.

## Materials and methods

### Human samples and SNPs genotyping

In total, 11 P2X7R SNPs were assessed on genomic DNA isolated from whole blood samples of both 1006 pancreatic cancer patients and 1673 controls. All procedures involving patients and patient materials were approved by the Danish Ethics Committee (see Ethical Approval). The Danish BIOPAC study “BIOmarkers in patients with PAncreatic Cancer (BIOPAC)—can they provide new information of the disease and improve diagnosis and prognosis of the patients?” (ClinicalTrials.gov ID: NCT03311776; (www.herlevhospital.dk/BIOPAC/) is a prospective multicenter open cohort study with ongoing enrollment. The control group consisted of healthy, post-menopausal Danish women included in the Danish Osteoporosis Prevention Study (DOPS, denoted Controls) [17]. Genotyping of both study groups was performed using TaqMan probes by LGC group (Hoddeston, UK). Furthermore, PDAC human cell lines used in the study were genotyped for P2X7R SNPs.

### Statistical analysis for SNPs identification

All statistics were performed using SPSS versions 20 and 28. Genotype distributions were tested for adherence to Hardy–Weinberg equilibrium (HWE) using the chi-square test. SNP genotype categories were recoded into dummy variables for SPS analysis as follows, homozygous wild type WT = 1, heterozygote HETE = 2 and homozygote HOMO = 3 (genotypes are given in Table 1). The distribution for all genotypes was compared with 1673 control cases from the Danish Osteoporosis Prevention Cohort (DOPS), Controls. The chi-square and Fisher’s exact tests were used to determine the differences in genotypic distribution between PDAC and Controls. Analysis of risk was performed as a case–control study, odds ratios (OR) and 95% confidence intervals (CI) were also calculated. Binary logistic regression analysis was used to correct for age and weight differences and diabetes. In all tests, p values less than 0.05 were considered statistically significant. Haplotype analysis was done by calculating pairwise linkage disequilibrium (LD) using Haploview version 4.0.

**Table 1:** P2X7R SNPs mutations in PDAC patients and controls

SNP	Genotype	Frequency (n and %)		Chi-square		Unadjusted OR			Adjusted (Age, Weight, Diabetes)		
		Control	PDAC	χ^2^	P	OR	CI (95%)	P	OR	CI (95%)	P
rs35933842 Null allele	GG	1479 (61.4)	889 (36.9)								
	GT	28 (1.2)	11 (0.5)	1.429	0.248	0.654	0.324–1.319	0.235	0.634	0.271–1.478	0.291
rs17525809 Val76Ala	TT	1327 (55.0)	771 (32.0)								
	TC	179 (7.4)	125 (5.2)			1.202	0.941–1.536	0.141	1.127	0.829–1.533	0.446
	CC	6 (0.2)	5 (0.2)			1.434	0.436–4.715	0.553	1.727	0.475–6.285	0.407
	TC + CC			2.392	0.134	1.209	0.950–1.539	0.122	1.149	0.851–1.553	0.364
rs28360447 Gly150Arg	GG	1458 (60.2)	876 (36.2)								
	GA	57 (2.4)	25 (1.0)			0.730	0.453–1.177	0.196	0.524	0.275–1.001	0.050
	AA	4 (0.2)	0								
	GG + GA			2.542	0.114	0.682	0.425–1.095	0.113	0.49	0.259–0.928	0.029
rs7958311 Arg270His	GG	840 (35.1)	527 (22.1)								
	GA	543 (22.7)	329 (13.8)			0.966	0.811–1.150	0.696	1.059	0.854–1.313	0.599
	AA	105 (4.4)	46 (1.9)			0.698	0.486–1.004	0.053	0.934	0.608–1.434	0.754
	GA + AA			0.894	0.348	0.922	0.780–1.091	0.344	1.040	0.847–1.277	0.709
rs7958316 Arg276His	GG	1462 (60.6)	869 (36.0)								
	GA	48 (2.0)	32 (1.3)	0.244	0.639	1.122	0.712–1.768	0.621	1.791	1.033–3.103	0.038
rs28360457 Arg307Gln	GG	1489 (61.3)	882 (36.33)								
	GA	37 (1.5)	21 (0.9)	0.024	0.892	0.958	0.557–1.647	0.877	0.993	0.513–1.920	0.982
rs1718119 Ala348Thr	GG	542 (22.7)	299 (12.5)								
	GA	719 (30.1)	434 (18.2)			1.094	0.910–1.316	0.340	1.075	0.857–1.348	0.534
	AA	235 (9.8)	160 (6.7)			1.234	0.965–1.578	0.093	1.141	0.842–1.546	0.396
	GA + AA			1.850	0.184	1.129	0.948–1.344	0.174	1.091	0.880–1.352	0.426
rs2230911 Thr357Ser	CC	1273 (52.5)	761 (31.4)								
	CG	239 (9.90)	134 (5.5)			0.938	0.745–1.180	0.584	0.785	0.588–1.048	0.101
	GG	12 (0.5)	5 (0.2)			0.697	0.245–1.986	0.499	0.672	0.193–2.336	0.531
	CG + GG			0.441	0.529	0.926	0.739–1.161	0.507	0.78	0.587–1.035	0.085
rs2230912 Gln460Arg	AA	1057 (43.6)	631 (26.1)								
	AG	415 (17.1)	245 (10.1)			0.989	0.821–1.191	0.907	1.041	0.830–1.306	0.729
	GG	46 (1.9)	28 (1.2)			1.020	0.631–1.648	0.937	0.744	0.394–1.407	0.364
	AG + GG			0.008	0.964	0.992	0.829–1.187	0.93	1.010	0.811–1.258	0.931
rs3751143 Glu496Ala	AA	1051 (43.5)	621 (25.7)								
	AC	432 (17.9)	246 (10.2)			0.964	0.801–1.160	0.696	0.866	0.688–1.090	0.221
	CC	38 (1.6)	29 (1.2)			1.292	0.789–2.115	0.309	1.716	0.970–3.037	0.064
	AC + CC			0.012	0.927	0.990	0.828–1.184	0.915	0.929	0.745–1.158	0.513
rs1653624 Ile568Asn	TT	1419 (58.6)	848 (35.0)								
	TA	99 (4.1)	54 (2.2)			0.913	0.648–1.285	0.601	1.084	0.712–1.649	0.707
	AA	2 (0.1)	0								
	TA + AA			0.409	0.549	0.895	0.636–1.258	0.523	1.064	0.700–1.616	0.773

Genotype frequencies are shown as absolute numbers and in percentages. Analysis depicts chi-square (χ^2^) and P values (P), unadjusted and adjusted odds ratios (OR), confidence intervals (CI) and P value (P). PDAC refers to pancreatic ductal adenocarcinoma

### Cell lines and chemicals

In this study, we used a pancreatic ductal adenocarcinoma cell line, PANC-1 (ATCC, Manassas, VA, USA, CRL-1469) (RRID:CVCL_0480), a human pancreatic stellate cell line, RLT-PSC (PSC)[32] (RRID:CVCL_A5TG), and for control HEK293 cells (ATCC, Manassas, VA, USA, CRL-1573) (RRID:CVCL_0045). Authenticity of cell lines was confirmed using STR profiling and were mycoplasma-free. Cells were grown in high glucose (25 mM) Dulbecco’s modified Eagle’s medium (DMEM GlutaMAX supplement, Thermo Fisher Scientific, Tåstrup, DK, 31,966–047) supplemented with 10% fetal bovine serum (FBS) and incubated at 37 °C with 5% CO_2_. P2X7R function was studied using the specific allosteric inhibitor AZ10606120 (Tocris, Bristol, UK, 3332/10), the competitive inhibitor A438079 (Tocris, Bristol, UK, 2972/10) and the agonist 2′(3′)-O-(4-Benzoylbenzoyl) adenosine 5′-triphosphate triethylammonium salt, BzATP (Sigma, Søborg, DK, 112898-15-4) and ATP (Sigma, Søborg, DK, A9187-1G). Antagonists were dissolved in DMSO (0.1%), agonists were dissolved in physiological buffer and adjusted to pH 7.4.

### P2X7R WT and SNP constructs, transfection and cell morphology

PANC-1, PSCs and HEK-293 cells have been transfected with 2–3 μg/ml of the construct encoding for P2X7R variants. The vector used in this study was pcDNA1.3 + and the hP2X7R gene is flanked by the Nhel and EcoRV restriction sites. The WT gene and P2X7R gene contain His155. The plasmid with the gene and with/without the mutation of interest was separated by an IRES sequence from GFP sequence, and therefore P2X7R and GFP were expressed simultaneously but separated. Thus, the cells containing and expressing the vector were GFP-positive. Transient transfection has been performed before each experiment using FuGENE HD Transfection reagent (Promega, E2311), ratio 1:3. We used green fluorescence to distinguish the transfected (P2X7R + GFP) from non-transfected cells expressing the native receptor. Overall cell morphology was assessed by imaging of cells (λ_ex_ = 588 nm, λ_em_ = 510 nm) in Leica TCS SP5 X confocal microscope (Leica Microsystems, Heidelberg, DE). The expression of the constructs was evaluated by Western blot. Primary antibodies were against the P2X7R (APR-004, Alomone Labs, Jerusalem, IL, RRID: AB_2040068), GFP (SC-8334, Santa Cruz, Tilst, DK, RRID:AB_641123), β-Actin (Sc-47778, Santa Cruz, Tilst, DK, RRID: AB_626632) and secondary antibody was HRP-conjugated (EZ-ECL-Biological Industries, Fredensborg, DK) and visualized with Fusion FX (Vilber Lourmat, Eberhardzell, DE).

### Calcium responses

PANC-1, PSCs and HEK293 cells were seeded in Wilco dishes and incubated with 5 μM Fura-2 AM (Invitrogen) for 40 min in physiological buffer without bicarbonate, denoted -BIC (mM): 140 NaCl, 1 MgCl_2_·6H_2_O, 1.5 CaCl_2_·2H_2_O, 0.4 KH_2_PO_4_, 1.6 K_2_HPO_4_·3H_2_O, 25 glucose and 10 HEPES, pH 7.4). Experiments were conducted at 37 °C on Nikon Eclipse Ti microscope. The cells were stimulated with BzATP and subsequently with the positive control ionomycin (1 μM). Drugs were added gently and directly to the non-perfused bath to minimize the effect of mechanical disturbance on ATP release, as detailed earlier [30]. P2X7R + GFP cells were identified with λ_ex_ = 470 nm and λ_em_ = 520 nm. Fura-2-was excited at λ_ex_ = 340 nm and λ_ex_ = 380 nm using a TILL Polychrome monochromator. Emission was collected at 510 nm with 40X oil objective (NA = 1.30) by an EMCCD camera (Andor X3 897, Belfast, UK) and digitized by a FEI image processing system (Thermo Fischer Scientific). The overlap of the image λ_ex_ = 470 allowed us to distinguish the P2X7R + GFP cells and the CTR (non-transfected) ones. The intracellular Ca^2+^ responses were recorded as the emission ratio of Fura-2 at 340/380 nm. The responses to the agonist were quantified as the area under the curve (AUC) and the peak response to the agonist, given as the ΔFura-2 (peak - baseline).

### YO-PRO-1 dye uptake

The uptake of high molecular weight molecules through P2X7R was determined by monitoring the uptake of YO-PRO-1, an impermeable dye that enters the cells through pores and binds the DNA emitting green fluorescence. Transfected PANC-1, PSCs and HEK293 cells were seeded in a 35 mm dish and incubated at 37 °C for 24 h. The following day, cells were washed with -BIC, and allowed to rest at 37 °C without CO_2_ in the Nikon Biostation IMQ. Time-lapse phase-contrast (Ph) and fluorescence (Fl) pictures were taken with 10X objective (NA = 0.5) for up to 3 h. YO-PRO-1 (2.5 µM) was added 12 min before stimulation with 5 mM ATP. Fluorescence intensity was analyzed for the same number of P2X7R + GFP and non-transfected cells using NIS Elements imaging software.

### Cytokines release

Media from transfected PSC cells was assayed for several released cytokines (IL-6, IL-1β, IL-8 and TNF-α) using V-PLEX Human Proinflammatory II (4-Plex) kit (Meso Scale, MSD MULTI-SPOT Assay System, K15053D-1). In addition, cells were washed and lysed and intracellular cytokine concentrations were measured and normalized to total protein extract.

### Cell migration

Transfected PANC-1 cells were seeded in a 35 mm dish coated with collagen I (8 µg/cm^2^) and allowed to rest for 24 h at 37 °C. Then the cells were washed, media replaced, and allowed to rest for 30 min. Time-lapse images were recorded in the Nikon Biostation IMQ with 20X objective (NA = 0.5) every 10 min for 11 h. Cells were stimulated with P2X7R agonist BzATP 100 µM 30 min after the starting point. Cells were tracked and the accumulated migration distance was calculated with ImageJ (version 1.47, National Institute of Health, Bethesda, MD).

### Statistical analysis for cell assays

GraphPad Prism 9.5.1 (GraphPad Software, San Diego, USA) was used for statistical analyses. All datasets were tested for normal distribution. Parametric data were tested with one-way ANOVA with subsequent Bonferroni correction. Comparisons between two specific conditions have been performed with two-tailed unpaired t test. Non-parametric data were analyzed with Kruskal–Wallis test and Mann–Whitney test for two groups. Normalized data were analyzed with appropriate test.

## Results

### Study population and P2X7R genotypes

The original population consisted of 1006 participants, aged 58–75 years at the time of inclusion in the BIOPAC study (see Methods). Among them, 11 cases were operated due to a tumor in pancreas, but the histological analysis showed that it was not cancer. 90 cases had other types of pancreatic cancer than adenocarcinoma, as judged by histology, and therefore they were excluded from the analysis. Thus, the study population (PDAC group) included 905 patients (410 women and 495 men), and since sex has minimal impact on the development of PDAC [2, 33], we used the data from 1673 women, aged 58–65 years, and previously collected and analyzed by Jørgensen et al. in the DOPS study (see Methods), as a control group [17]. The study population was genotyped for 11 non-synonymous SNPs in *P2RX7*. Additional file 2: Table S1 shows the predicted amino acid change and minor allele frequency in both PDAC and control group and adherence to Hardy–Weinberg equilibrium (HWE). No HWE disequilibrium was detected except for one mutation (Glu496Ala). A chi-square test was performed for genotype distribution in PDAC patients compared to controls. However, we did not find any genotype that showed significant differences (Additional file 2: Table S1), indicating that there is no difference in genotype distribution between PDAC and control groups.

### P2X7R haplotype analysis

Pairwise linkage disequilibrium (LD) was calculated using Haploview version 4.0 and a diagram is shown in Additional file 1: Fig. S1. A haplotype block was found to include four base positions: G1068A, C1096G, A1405G and A1513G. Four common haplotypes comprising 99.92% of all haplotypes were found. These were designated haplotype 1 (G1068-C1096-A1405-A1513) accounting for 33.8% of the alleles, haplotype 2 (A1068-C1096-A1405-A1513) accounting for 25.4% of the alleles, haplotype 3 (G1068-C1096-A1405-C1513) accounting for 16.6% of the alleles, haplotype 4 (A1068-C1096-G1405-A1513) accounting for 16.2% of the alleles. This haplotype block overlaps with the previously published haplotype [17, 34]. In addition, we found a strong pair-wise linkage disequilibrium between 474G>A (Gly150Arg) in the haplotype block in control subjects but not in PDAC patients.

### Two SNP variants, Gly150Arg and Arg276His, have different risk association in PDAC

**Fig. 1:**
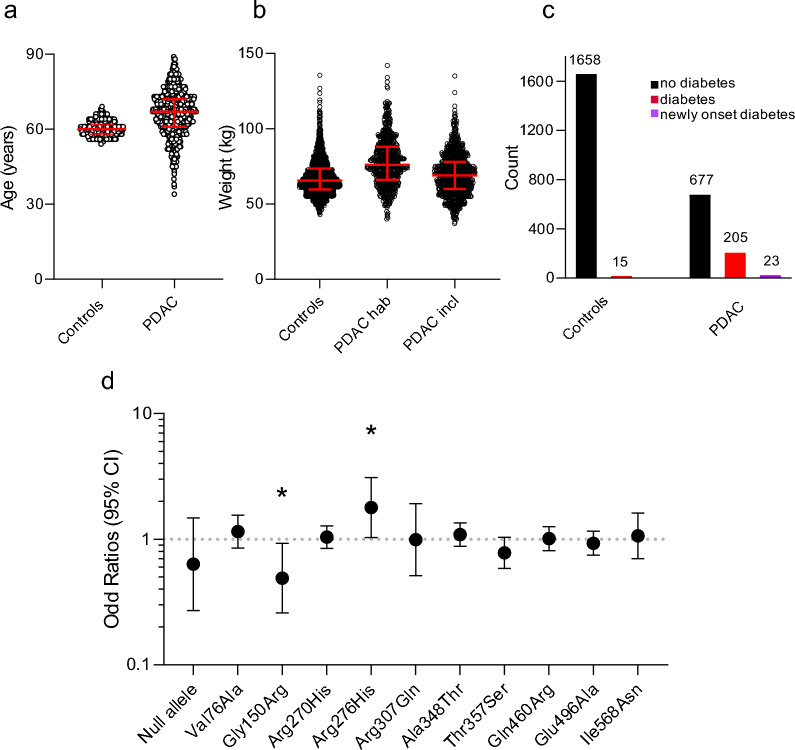
Distribution of the variables for the PDAC patients and controls included in OR analysis. **a** Age distribution, PDAC patient and controls. **b** Weight distribution for PDAC patients: habitual weight and the weight at the time of inclusion. The data refer to patients: 638 (habitual weight), 842 (weight at inclusion) and 1673 (controls). **c** Number of PDAC patients and controls without or with diabetes (Type 2, possibly some Type 1) at the time of inclusion (diabetes) or as a consequence of PDAC (newly onset diabetes). Parts a, b show the median and the interquartile in red; part c shows the numbers of patients. **d** Adjusted OR and 95% confidence interval for 11 SNP analyzed and detailed in Table 1. Green bars indicate 2 SNPs with statistical significance *p < 0.05

The incidence of pancreatic cancer increases with age and recent studies indicate association of PDAC with obesity and diabetes [35, 36]**.** Moreover, patients lose weight and often develop diabetes during the disease progression [37]. The distribution of our study population in relation to age, weight and diabetes is reported in Fig. 1a–c. For further analysis, since we did not have a sufficient number of heights recorded, we did not include BMI, but patient weight at inclusion. For diabetes, we included all patients receiving treatment for diabetes at the inclusion, as the exact type of diabetes was not recorded. Analysis of risk was performed on the control group and cancer group using a binary logistic regression analysis without correction or with adjustment for variables in our study—age, weight, and diabetes. Unadjusted and adjusted odds ratio values (OR) are reported in Table 1, with respective p values. After correction, two SNP variants showed significant risk association with PDAC as shown in Table 1 and Fig. 1d. The first one is 474G>A (Gly150Arg), which was associated with a decreased risk of PDAC, with adjusted OR = 0.490 (p = 0.029). The second is 853G>A (Arg276His), which was associated with an increased risk for PDAC, with adjusted OR = 1.791 (p = 0.038).

### Expression of P2X7R SNPs and WT in pancreatic cancer and stellate cell

**Fig. 2:**
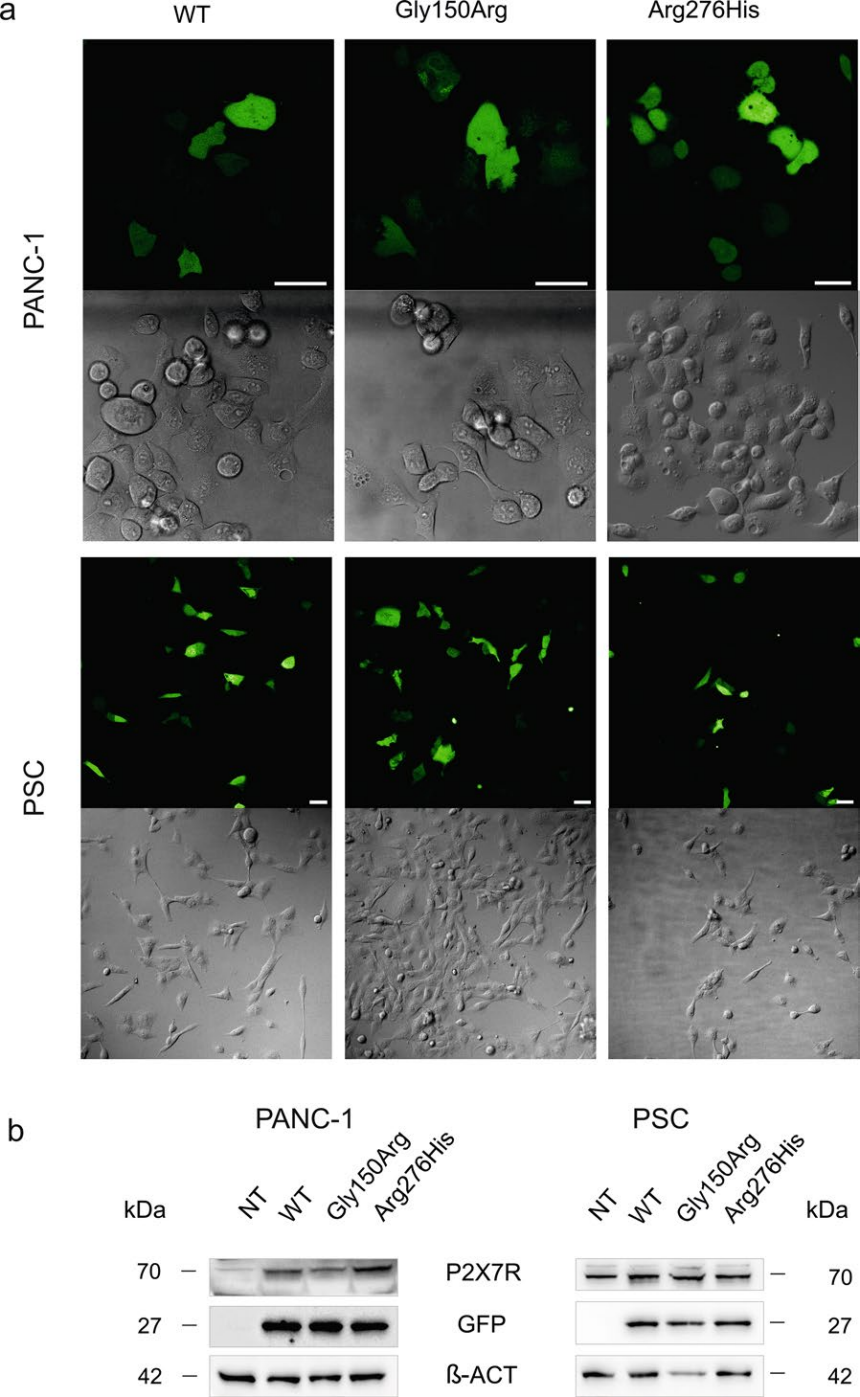
Cell morphology and P2X7R expression in PANC-1 and PSC cells. **a** Representative fluorescence and transmission images of cells expressing P2X7R + GFP variants. Bars are 50 µm. **b** Representative WB on whole cell lysate showing P2X7R expression in transfected and non-transfected (NT) cells. GFP and β-actin are also shown as transfection and loading control, respectively

We wished to express relevant SNPs in human pancreatic cancer and in stellate cells and therefore we screened several common pancreatic cancer cell lines for P2X7R SNPs to check that they did not express the relevant SNP variants. We found that they all contain at least one P2X7R SNP variant (Additional file 2: Table S2). We focused on PANC-1 cells that express both 1068G>A (rs1718119, Ala348Thr) and 1405A>G (rs2230912, Gln460Arg) variants resulting in GOF and LOF, respectively. We further chose PSC cells (RLT-PSC) which express the 1068G>A variant. We chose these cells because they have been best characterized with respect to the endogenously expressed P2X7R and its effects [28, 30, 31], therefore, we judged them to be adequate model systems. We over-expressed WT and SNPs in PANC-1 and PSCs and visualized them with GFP using live-cell imaging in a confocal microscope. Figure 2a shows that in both cell types, the expression of the different P2X7R variants did not change apparent cell morphology. Figure 2b shows the expression of P2X7R proteins compared to GFP and β-actin and results indicate robust and similar expression of WT and SNP-containing receptors in our cells lines.

### ***Ca***^***2***+^***dynamics show SNP-specific differences in PANC-1 and PSCs***

**Fig. 3:**
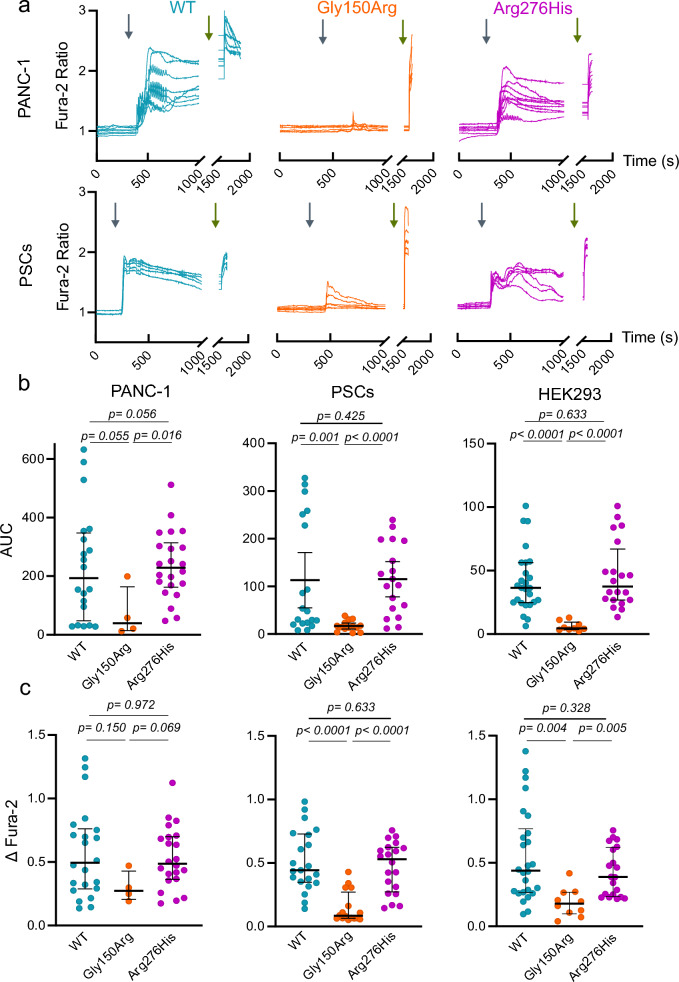
Effect of P2X7R SNPs on calcium transients in PANC-1, PSCs and HEK293. **a** Representative recordings of Ca^2+^ signals (Fura-2 ratio) in P2X7R + GFP PANC-1 and PSCs (top and bottom panels) transfected with WT (blue), Gly150Arg (orange) and Arg276His (magenta) receptor. The graphs show the response after stimulation with BzATP 100 µM (grey arrow) and the positive control ionomycin 1 µM (green arrow). **b** Area under the curve (AUC) calculated for 600 s (PANC-1), 400 s (PSCs) and 160 s (HEK293) after the response starts. **c** Change in Fura-2 (ΔFura-2) between the basal and the maximum peak response to the agonist. Each point represents the change of Fura-2 in a single cell. Comparison between two variants have been evaluated with the non-parametric Mann–Whitney test and the p values are reported in the graphs. Each graph in (**b**, **c**) shows responses of cells in 3–4 independent experiments and medians and the interquartile range

P2X7R is an ion channel that, once activated with ATP, allows the flux of cations, including influx of Ca^2+^. Here, we monitored intracellular Ca^2+^ responses using Fura-2 in PANC-1 and PSCs expressing the three variants of P2X7R denoted as WT, Gly150Arg and Arg276His. Cells were stimulated with the ATP agonist BzATP (100 µM), which has tenfold higher apparent affinity than ATP [38]. Additional file 2: Table S3 shows percentages of responding cells in cells transfected with P2X7R + GFP variants. Note that in the Gly150Arg variant the number of responding cells is low. Focusing on P2X7R + GFP transfected cells, our results show interesting differences in Ca^2+^ signals depending on the construct expressed. Ca^2+^ responses of PANC-1 and PSC cells are shown in Fig. 3a, and a summary of peak responses (ΔFura-2) and AUC are given in Fig. 3b, c. In both PANC-1 and PSC cells expressing Arg276His variant resulted in similar Ca^2+^ responses upon BzATP stimulation compared to WT. In contrast, transfection of the Gly150Arg variant resulted in significantly lower or undetectable responses (Fig. 3a), as reflected by a lower number of responding cells (Additional file 2: Table S3). The Ca^2+^ data obtained in PDAC cell lines are consistent with the ones obtained in HEK293 cells transfected with the same constructs (Fig. 3).

### Gly150Arg and Arg276His variants affect dye uptake in PANC-1 and PSCs

**Fig. 4:**
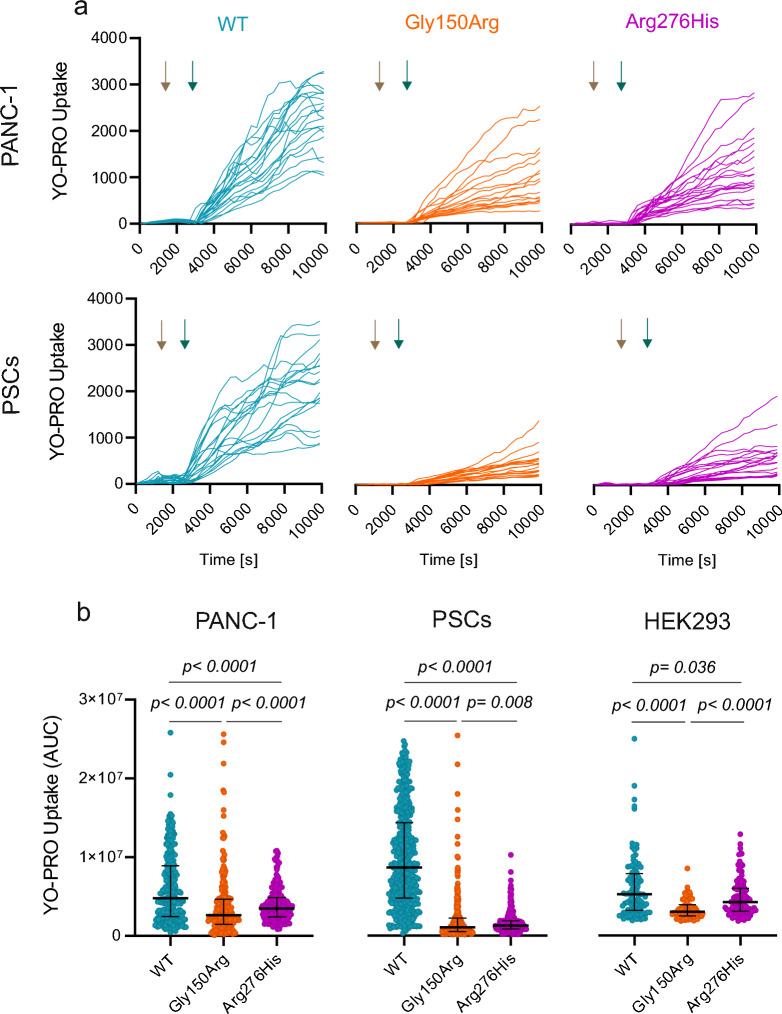
Reduced ATP-induced dye uptake in PANC-1, PSCs and HEK293 cells expressing Gly150Arg and Arg276His. **a** Original single-cell traces of the ATP-induced YO-PRO-1 uptake in P2X7R + GFP PANC-1 (top) and PSCs (bottom) expressing WT (blue), Gly150Arg (orange) and Arg276His (magenta) receptors. YO-PRO-1 was added after 25 min (beige arrow) prior to stimulation with 5 mM ATP (green arrow). **b** Comparison of AUC of P2X7R + GFP expressing the different P2X7R variants. Statistical significance was evaluated with the non-parametric Mann–Whitney test and the p values are reported in the graphs. Graphs in (**b**) and (**c**) include results from single cells obtained in 3–4 independent experiments

To study macropore properties associated with P2X7R, we monitored the uptake of fluorophore YO-PRO-1 in cells overexpressing WT receptor or Gly150Arg and Arg276His after stimulation with 5 mM ATP (Fig. 4a). The summarized data show that overexpression of P2X7R-WT significantly increased the YO-PRO-1 uptake in P2X7R + GFP cells compared to non-transfected cells in both PANC-1, PSCs and HEK293 cells (Additional file 1: Fig. S2). The impact of P2X7R-WT overexpression was higher on PSCs. Figure 4b shows that expression of P2X7R + GFP with Gly150Arg reduced dye uptake compared to WT to about 55% in PANC-1 cells and to about 12% in PSC cells. Expression of P2X7R + GFP Arg276His also reduced dye uptake, but the effect was less pronounced, especially in PANC-1 cells, where dye uptake was reduced to about 73% while in PSC cells it was reduced to 15% (Fig. 4b). In HEK293 cells, the expression of P2X7R + GFP with Gly150Arg or Arg276His reduced dye uptake to 58% or 81%, respectively. Overall, expression of Gly150Arg significantly reduced the dye uptake in all cell types, while the reduction upon expression of Arg276His was most prominent in PSCs compared with PANC-1 and HEK293 cells.

### Minor effect of P2X7R SNP variants on PANC-1 and PSCs survival

Our previous work on the role of P2X7R in PDAC showed its involvement in PANC-1 and PSCs proliferation and cell death (see Introduction). Therefore, we studied CCK-8 incorporation and LDH release in the same cell lines, transiently expressing each of the three constructs of interest and results are shown in Additional file 1: Fig. S3. Overall, in both cell types, the P2X7R seems not to be detrimental for cell survival in a population that includes transfected and non-transfected cells, at least under the assay conditions used here. Note that in basal conditions (no ATP or BzATP added) P2X7R is already activated, probably due to ATP present or released into the media, and therefore P2X7R inhibition has effects as reported earlier [28, 30, 31].

### Cytokine release in PSCs

**Fig. 5:**
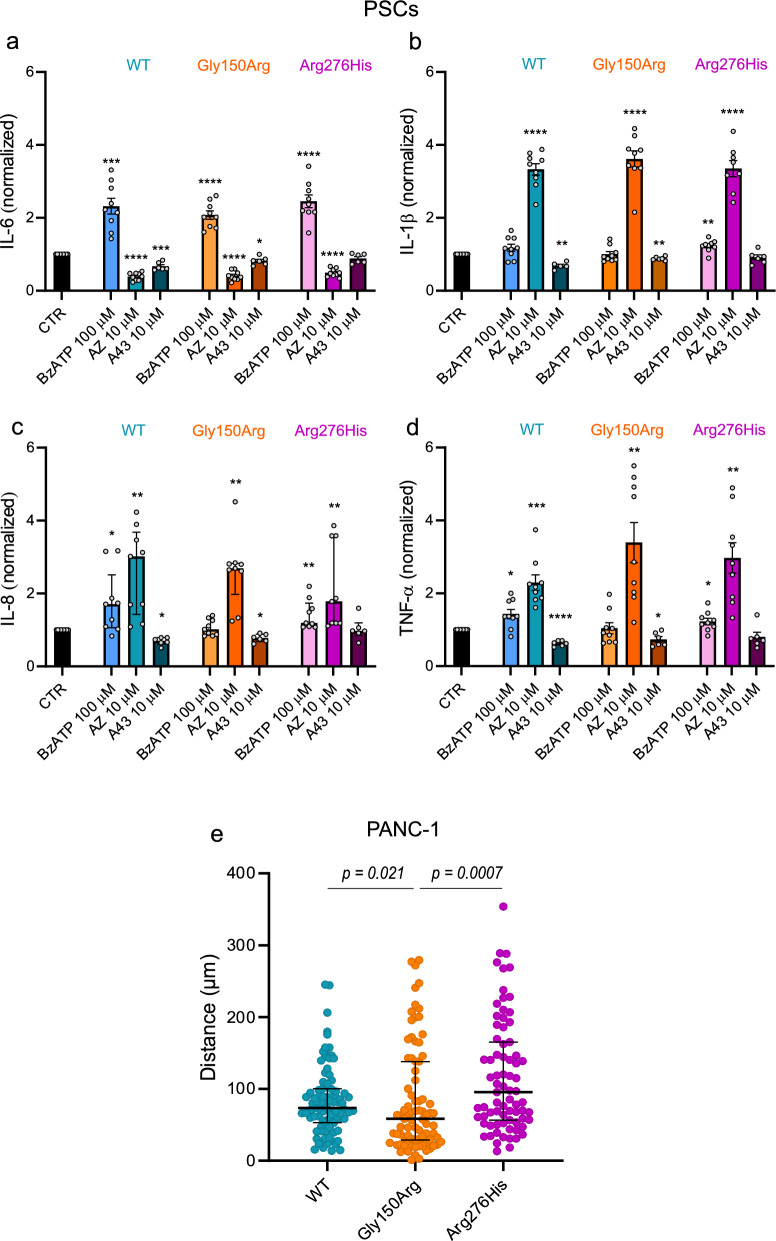
Effects of the P2X7R variants on cell-specific functions: cytokine release from PSCs (**a**–**d**) and migration of PANC-1 cells (**e**). **a**–**d** IL-6, IL-1β, IL-8 and TNF-α released in the supernatant of PSCs expressing WT (blue), Gly150Arg (orange) and Arg276His (magenta) receptors. Stimulation with BzATP 100 µM and inhibition with AZ10606120 (AZ) 10 µM and A438079 (A43) 10 µM are compared to the respective unstimulated controls cells (CTR). The data are shown as the mean ± SEM for IL-6, IL-1β and TNF-α, and median ± interquartile range for IL-8, of n = 9. **e** Migration of P2X7R + GFP PANC-1 expressing P2X7R variants**.** Significance has been evaluated as followed: IL-6, IL-1β and TNF-α statistics have been performed with one sample t test, and reported as *p < 0.05; **p < 0.01; ***p < 0.001; ****p < 0.0001; IL-8 statistics have been performed with one-sample Wilcoxon signed rank test and reported as *p < 0.05; **p < 0.01.  Migration has been analyzed with the non-parametric Mann–Whitney test and the p values are reported in the graph

Our previous work showed that P2X7R causes IL-6 release in PSCs. In the present study, we confirm this finding by showing that BzATP increased IL-6 release in all transfected cells compared to the basal (CTR) state (Fig. 5a). The P2X7R inhibitor AZ10606120 (10 µM) significantly reduced IL-6 release (Fig. 5a). In contrast to the effects on IL-6, for other cytokines (IL-1β, IL-8 and TNF-α) BzATP had small stimulatory effects on WT and Arg276His variants, but not on Gly150Arg (Fig. 5b–d). Most remarkably though, AZ10606120 increased cytokine IL-1β, IL-8 and TNF-α release in WT and the two SNPs. Therefore, we tested another P2X7R inhibitor A438079, which had the predicted effect by inhibiting release of IL-6 and other cytokines in WT and the Gly150Arg variant, although not in the Arg276His variant. In addition to the assay on cytokine release, we also analyzed intracellular cytokine levels (cell lysates), with the data shown in Additional file 1: Fig. S4. Again, AZ10606120 had pronounced stimulatory effects, especially on IL-1β and IL-8.

### Role of P2X7R in PANC-1 migration

P2X7R promotes cancer cell migration in PDAC [28], hence we analyzed migration of PANC-1 transfected with each of the three different constructs (Fig. 5e). The data indicate that the Gly150Arg variant expressing cells migrated shorter distances than cells expressing WT and Arg276His variants.

## Discussion

In this study, we analyzed the occurrence of 11 non-synonymous SNPs in *P2RX7* in DNA extracted from blood samples of PDAC and control individuals and correlated them with the risk of developing the disease. We show that two P2X7R SNP variants are associated with opposite risks of developing PDAC. Functional studies of the P2X7R-WT and SNPs in pancreatic cancer and stellate cells reveal some potentially relevant differences and below we discuss whether these could help us to understand their role in pancreatic cancer.

In PDAC, apart from four key driver genetic mutations (*KRAS*, *CDKN2A/p16*, *TP53*, *SMAD4*), other mutations in inherited susceptibility genes, e.g. BRCA2, MLH1, are risk factors for pancreatic cancer [3]. Since the P2X7R plays an important role in cell and animal models of PDAC [7, 28–31], and because an increasing number of studies point to an association of SNPs in P2X7R and human disease, it was timely to investigate whether P2X7R SNPs are differentially expressed in PDAC patients. First, we analyzed whether certain SNPs occurred with higher frequency in PDAC patients in comparison to control individuals. We could not detect any significant difference in the frequency of P2X7R SNPs, which is possibly due to a low number of samples or a result of the relatively frequent occurrence of P2X7R variants. However, correlation analysis with the risk of PDAC, showed significance for two SNP variants that occur in the P2X7R extracellular domain, i.e. Gly150Arg with OR = 0.490 and Arg276His with OR = 1.791, which are respectively associated with decreased and increased risk of PDAC (Table 1). In this analysis, we assumed that sex and ethnicity do not have significant impact on PDAC incidence, while age, weight and diabetes do [2, 35, 36], and out OR were adjusted for these parameters. Further studies with larger and more varied control and PDAC groups would be of value.

On the cellular level, P2X7R SNPs have been studied mainly in HEK293 cells by monitoring effects on macropore formation and ionic currents [16, 34]. The Gly150Arg variant causes greatly diminished dye uptake and complete lack of currents, thus classifying it as a LOF variant [16]. We confirm the findings on dye uptake (Fig. 4). Regarding Arg276His, only one study on HEK293 cells has been published, where markedly reduced dye uptake was reported, thus denoting it is also LOF variant [34]. We find that this variant had relatively small reduction on dye uptake (Fig. 4). Nevertheless, to evaluate the role of SNPs in PDAC, we reasoned that it would be more appropriate to study the functionality of SNP variants in more relevant cells, in particularly pancreatic cancer (PANC-1) and stellate cells (PSCs). Cells transfected with WT or SNP variants (and GFP) showed no obvious change in cell morphology compared to cells with the native receptor (Fig. 2).

Assaying the dye uptake (Fig. 4), we found that both SNP variants, Gly150Arg and Arg276His, impair YO-PRO-1 uptake in both cell lines in comparison to WT cells, though Gly150Arg variant has more pronounced effects than Arg276His one (Fig. 4). However, the impact of the two SNP variants was quite different in experiments monitoring Ca^2+^ responses (Fig. 3). In cancer cells, Gly150Arg variant resulted in non-responsive cells or cells showing less pronounced responses than WT-expressing cells, thus displaying clear traits of LOF. However, Arg276His variant showed WT-like Ca^2+^ signals. Similar results were obtained in PSCs.

Decreased function in cells expressing Gly150Arg (decreased dye uptake and calcium signals) is consistent with the published data as a LOF variant [16, 34]. Gly150 is a highly conserved residue in P2X7R in different species and it may contribute to structural flexibility important for the gating of the channel [16]. The Gly150Arg variant might impact receptor function by introducing a large positively charged side chain. However, in cells expressing the Arg276His variant, we observed disparate effects on functionality. While dye uptake was reduced, intracellular Ca^2+^ responses were largely comparable to the WT receptor. Therefore, we performed these assays also in HEK293 cells and found that Ca^2+^ responses were similar to those obtained in PANC-1 cells and PSCs (Fig. 3). In the murine P2X7R, the Arg276 locus has been associated with the receptor ATP binding site, and the variant shows enhanced ATP sensitivity [8, 39]. As there are differences between the murine and the human receptor, we cannot conclude whether this is also valid for Arg276 in the human receptor and in particular for the Arg276His variant [38].

Regarding cancer cell migration, which is relevant to metastasis, we already know that endogenously expressed P2X7R promotes PANC-1 migration [28]. In the present study, our observation indicates that PANC-1 migration was slightly decreased in cells with the Gly150Arg variant (Fig. 5).

In single cell assays (dye uptake, calcium influx), we could visually monitor transfected cells. Longer-term assays performed on a population of cells (cell proliferation and cytotoxicity) were less sensitive and not greatly affected by the expression of P2X7R-WT or SNPs, especially PANC-1 cells that highly express endogenous P2X7R (Additional file 1: Fig. S3). Moreover, cell media contains ATP that can potentially activate the P2X7Rs [28, 30, 31]. Activation and inhibition of the P2X7R had similar effects on cell survival as reported for native receptors expressed in PANC-1 and PSC cells [28, 30, 31].

Pancreatic stellate cells release several factors that can affect cancer cells [30]. Interestingly, IL-6 has been identified as an inflammatory marker associating with increased one-year mortality in pancreatic cancer patients [40]. In our study, we show that stimulation of the P2X7R in PSCs with BzATP increased IL-6 release in all variants (Fig. 5a). AZ10606120 reduced IL-6 release in WT and two SNP variants while A438079 was also effective, though not in the Arg276His one. Regarding other cytokines (IL-1β, IL-8 and TNF-α), we obtained some intriguing findings. Activation by BzATP was relatively weak on all PSCs and it appears that we could not activate the Gly150Arg variant. In contrast, we could not inhibit Arg276His variant with A438079. Most paradoxical, AZ10606120, but not A438079, had a large stimulatory effect on the release of these three cytokines. First, one can suggest that this is a non-specific or off-target effect. Yet in relation to IL-6 release, AZ10606120 behaved as a P2X7R inhibitor. Moreover, AZ10606120 also increased cellular cytokine levels, and this seems to be most prominent in Arg276His variant (Additional file 1: Fig. S4). Assuming that AZ10606120 is a specific allosteric inhibitor of P2X7R, it may prevent loss of cellular K^+^, thereby hyperpolarize cells and improve protein/cytokine production. Since there is a remarkable inhibitory effect on IL-6 release, but stimulatory effect on release of the other three cytokines, these disparate effects indicate that there is coupling to different signaling and/or cytokine release machinery, or different extent of receptor activation by the given agonist concentration. For example, P2X7R interaction with pannexin-1 [41] could be relevant for IL-6 release, whereas the classical NLRP3 inflammasome activation and release via gasdermin or vesicular release could be an option for the other three cytokines [42, 43]. Therefore, although we know a lot about the effect of P2X7R inhibitors at the receptor/ion channel level, effects on cellular down-stream effects are yet to be resolved.

Taken together, our data show that the Gly150Arg variant displays a LOF phenotype (decreased pore and calcium signals in PANC-1 and PSC cells, decreased PANC-1 migration), which is consistent with the published data [16, 34]. However, in cells expressing Arg276His variant, we had disparate effects in functionality (minor decrease in dye uptake, but WT-like Ca^2+^ influx in both cell types and slightly increased migration). How could these phenotypes translate into altered risk of PDAC? LOF variants may protect the pancreas from inflammatory reactions, since inflammation/chronic pancreatitis are important risk factors [3, 44]. Several cell types are involved in inflammation including macrophages, and published data on washed blood show that Gly150Arg variant induced low level of dye uptake [45]. The Arg276His variant may have additional yet unknown downstream effects, and interestingly this variant is resistant to A438079-mediated inhibition of cytokine release. Therefore, we propose that the Gly150Arg variant is LOF and protective, while Arg276His is a potential risk factor in PDAC, although its precise phenotype is yet to be clarified.

For other diseases, the Gly150Arg variant has been associated with reduced bone mineral density and higher risk of osteoporosis [18, 46]; mood disorders [16]; impaired phagocytosis and subsequent aged related macular degeneration (when associated with P2X4R - Tyr315Cys) [47]; and risk of primary progressive multiple sclerosis [48]. Based on these findings and on the fact that Gly150Arg is considered a LOF variant, one could argue that normal P2X7R expression and function are likely to support a normal healthy state. In contrast to above, our study on PDAC indicates that Gly150Arg LOF is a protective variant, possibly due to lower inflammasome activation and inflammation in general and/or slower progression of pancreatic neoplasia. As for Arg276His, this variant is rarely tested in genotype studies [49]. Therefore, our study is important because it reports this variant to be associated with higher risk of pancreatic cancer.

Interestingly, a few other P2X7R SNP variants are reported for other cancers in association studies, as reviewed recently [15]. Most relevant for our study is the fact that diabetes and pancreatic cancer appear to be associated, not least because inflammation plays an important role in both [36, 37]. One study indicates that several P2X7R SNP variants appear to associate with altered glucose homeostasis [50] and another indicates that the Ala348Thr variant in type 2 diabetic patients is associated with increased insulin release, but not glycemic control [51]. In light of our finding that diabetes is one of the important co-variants in revealing P2X7R association in PDAC (Fig. 1), a closer examination of P2X7R SNPs in diabetes would be important.

## Conclusion

**Fig. 6:**
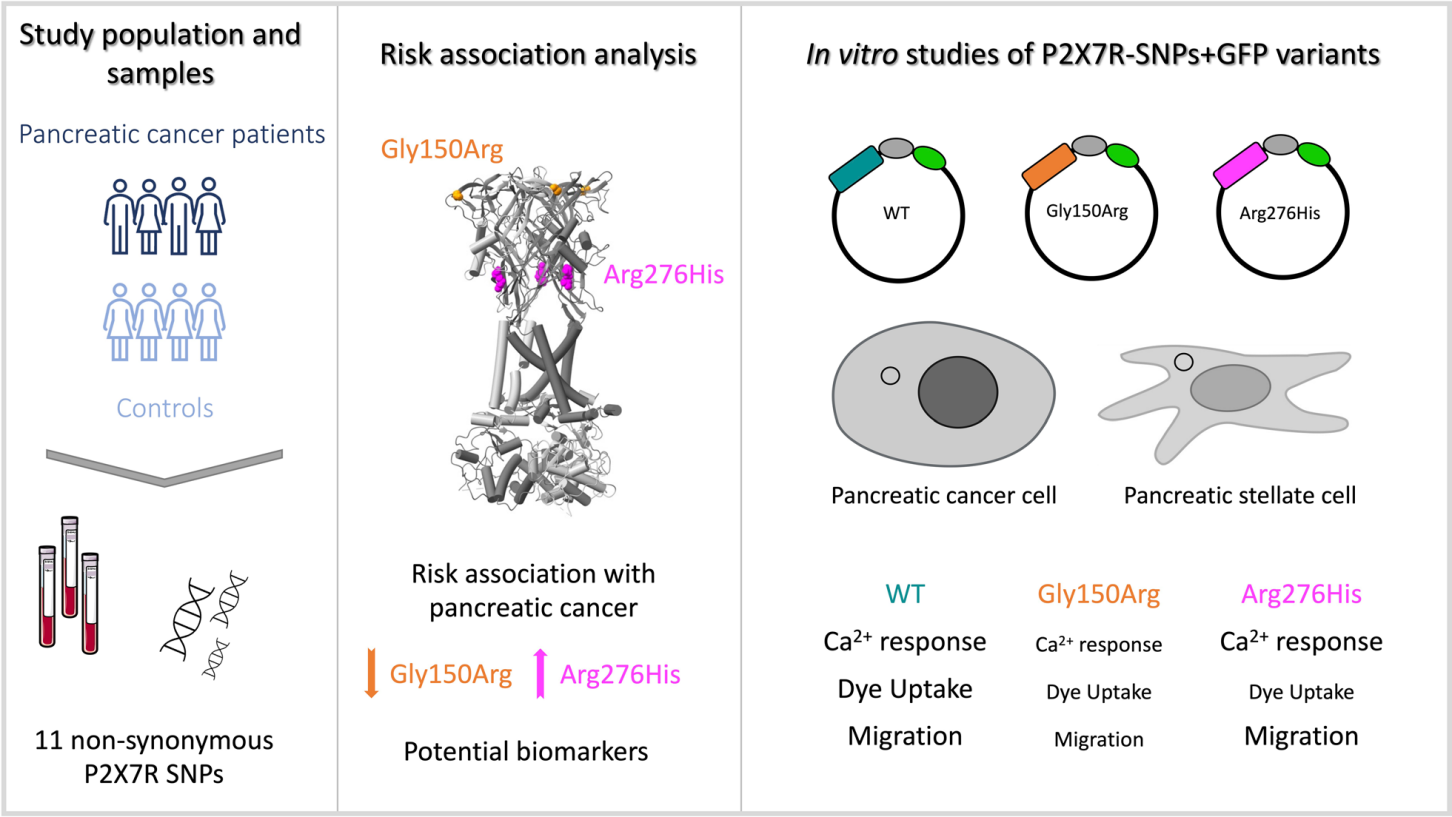
The graphical presentation of the study design and main findings

In conclusion, our data show that two SNP variants in P2X7R correlate with different probability of developing PDAC, which is partially supported also by our functional data on PDAC cells. SNP variant Gly150Arg displays LOF traits and correlates with a protective phenotype, while Arg276His is correlating with higher risk of developing pancreatic cancer and could be considered as a potential biomarker. The graphical presentation of the study design and main findings are displayed in Fig. 6.

## Supplementary Information

**Additional file 1**: **Fig. S1**. Haploview analysis of pairwise LD (linkage disequilibrium) between P2X7R polymorphisms; **Fig. S2** Increased ATP-induced dye uptake in PANC-1, PSCs and HEK293 over-expressing P2X7R+GFP WT compared to non-transfected cells (CTR); **Fig. S3**. Effect of P2X7R SNPs on cell survival; **Fig. S4**. Cytokines quantification in PSCs lysates.

**Additional file 2**: **Table S1** Frequency of common nonsynonymous P2X7R-SNPs; **Table S2** P2X7R-SNP analysis in pancreatic cell lines; **Table S3** Ca^2+^ signals in P2X7R-GFP cells.

## Data Availability

All data supporting the finding of this study are available within the paper and its Supplementary Material.

## Abbreviations

ATP: Adenosine triphosphate
AUC: Area under the curve
AZ10606120: N-[2-[[2-[(2-hydroxyethyl)amino]ethyl]amino]-5-quinolinyl]-2-tricyclo[3.3.1.13,7]dec-1-ylacetamide dihydrochloride
A438079: 3-[[5-(2,3-Dichlorophenyl)-1H-tetrazol-1-yl]methyl]pyridine hydrochloride
BzATP: 2′(3′)-O-(4-benzoylbenzoyl) adenosine 5′-triphosphate
-BIC: Physiological buffer without bicarbonate
BMI: Body mass index
CCK-8: Cell counting kit-8
DMSO: Dimethyl sulfoxide
ERK: Extracellular signal-regulated kinase
FBS: Fetal bovine serum
GFP: Green fluorescent protein
GOF: Gain-of-function
HEPES: 4-(2-Hydroxyethyl)-1-piperazineethanesulfonic acid
HWE: Hardy–Weinberg equilibrium
IL-1β: Interleukin 1β
IL-6: Interleukin 6
LD: Linkage disequilibrium
LDH: Lactate dehydrogenase
LOF: Loss-of-function
NA: Numerical aperture
NLRP3: NLR family pyrin domain containing 3
OR: Odds ratio
PDAC: Pancreatic ductal adenocarcinoma
PSC: Pancreatic stellate cell
P2X7R: The P2X7 receptor
SNP: Single nucleotide polymorphism
TME: Tumor microenvironment
TNFα: Tumor necrosis factor α
