# Supplementary material for: Human P2X7 receptor variants Gly150Arg and Arg276His polymorphisms have differential effects on risk association and cellular functions in pancreatic cancer
Source: Cancer Cell Int. 2024 Apr 25;24:148. doi: 10.1186/s12935-024-03339-9 (PMC11044319; doi:10.1186/s12935-024-03339-9)
Supplement: Supplementary file 1 — Additional file 1: Fig. S1. Haploview analysis of pairwise LD (linkage disequilibrium) between P2X7R polymorphisms; Fig. S2 Increased ATP-induced dye uptake in PANC-1, PSCs and HEK293 over-expressing P2X7R+GFP WT compared to non-transfected cells (CTR); Fig. S3. Effect of P2X7R SNPs on cell survival; Fig. S4. Cytokines quantification in PSCs lysates. [file 12935_2024_3339_MOESM1_ESM.pdf]

Supplementary Figures

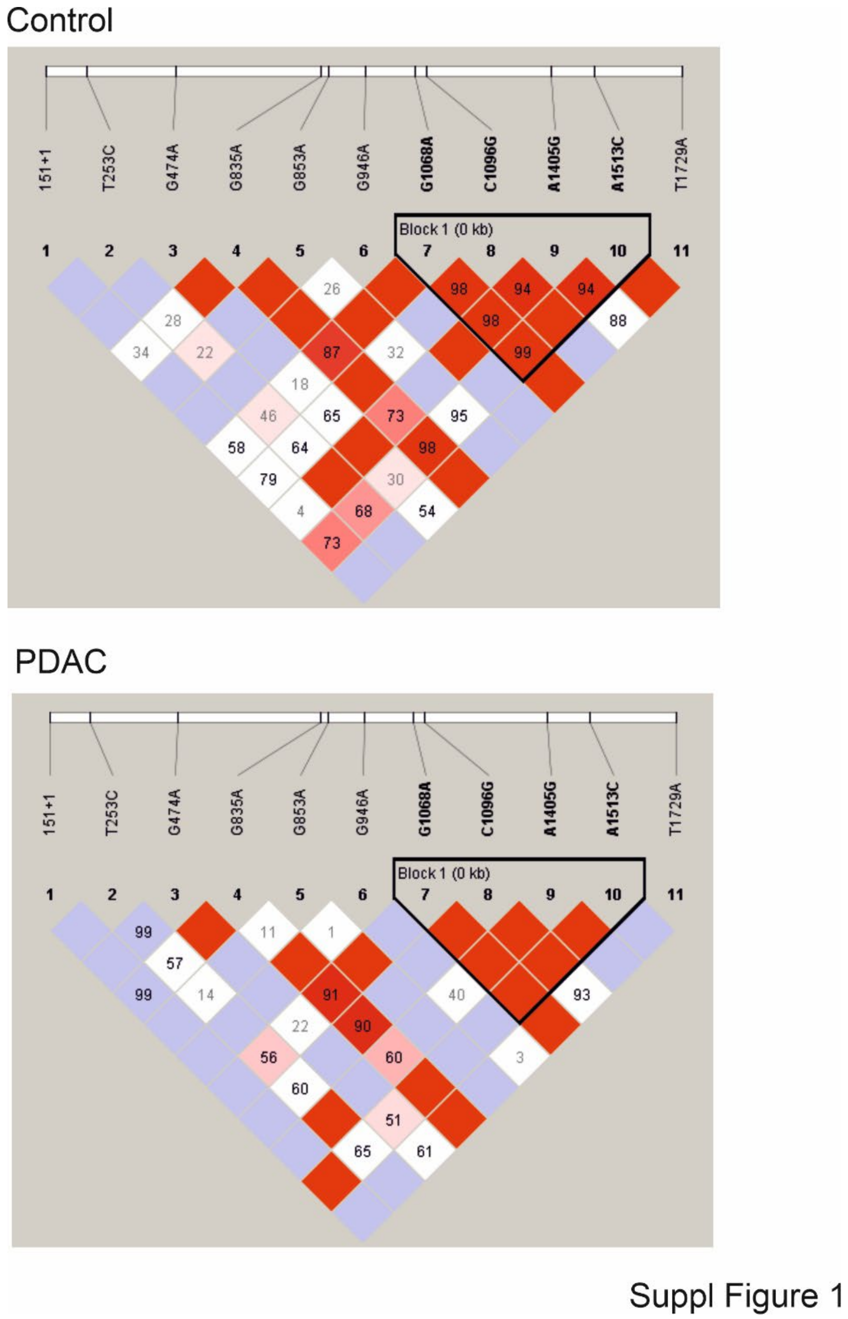

Figure S1. **Haploview analysis of pairwise LD (linkage disequilibrium) between P2X7R polymorphisms.** Each diamond represents a pairwise LD relationship between two SNPs, the numbers stated in the diamonds state for D' value multiplied by 100. Diamonds without a number indicate D'=1. The colors represent the relative D'/LOD (log of the odds) score where bright red is D'=1, LOD ≥2; blue is D'=1, LOD<2; different shades of pink is D'<1, LOD ≥2; white is D'<1, LOD<2.

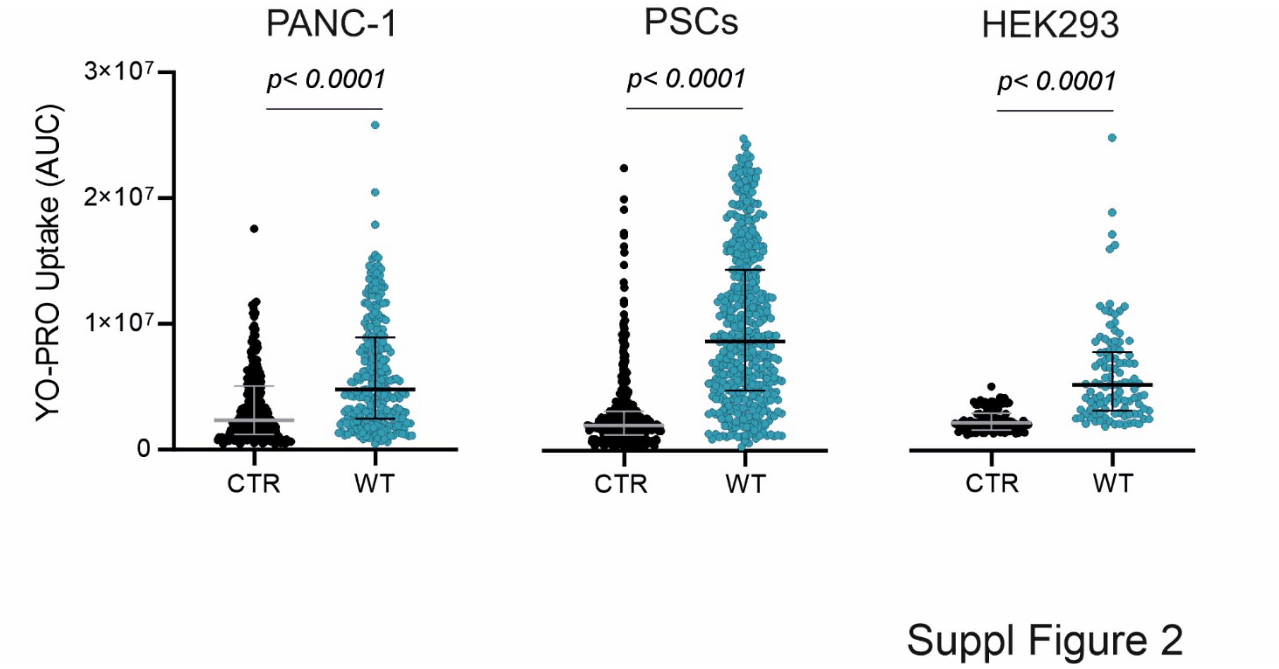

Figure S2 Increased ATP-induced dye uptake in PANC-1, PSCs and HEK293 over-expressing P2X7R+GFP WT compared to non-transfected cells (CTR). Area under the curve (AUC) of P2X7R+GFP (blue) WT transfected cells compared to non- transfected (black) in non-transfected cells. Statistical significance was evaluated with the non-parametric Mann-Whitney test and the p-values are reported in the graphs. Graphs include results from single cells obtained in 3-4 independent experiments.

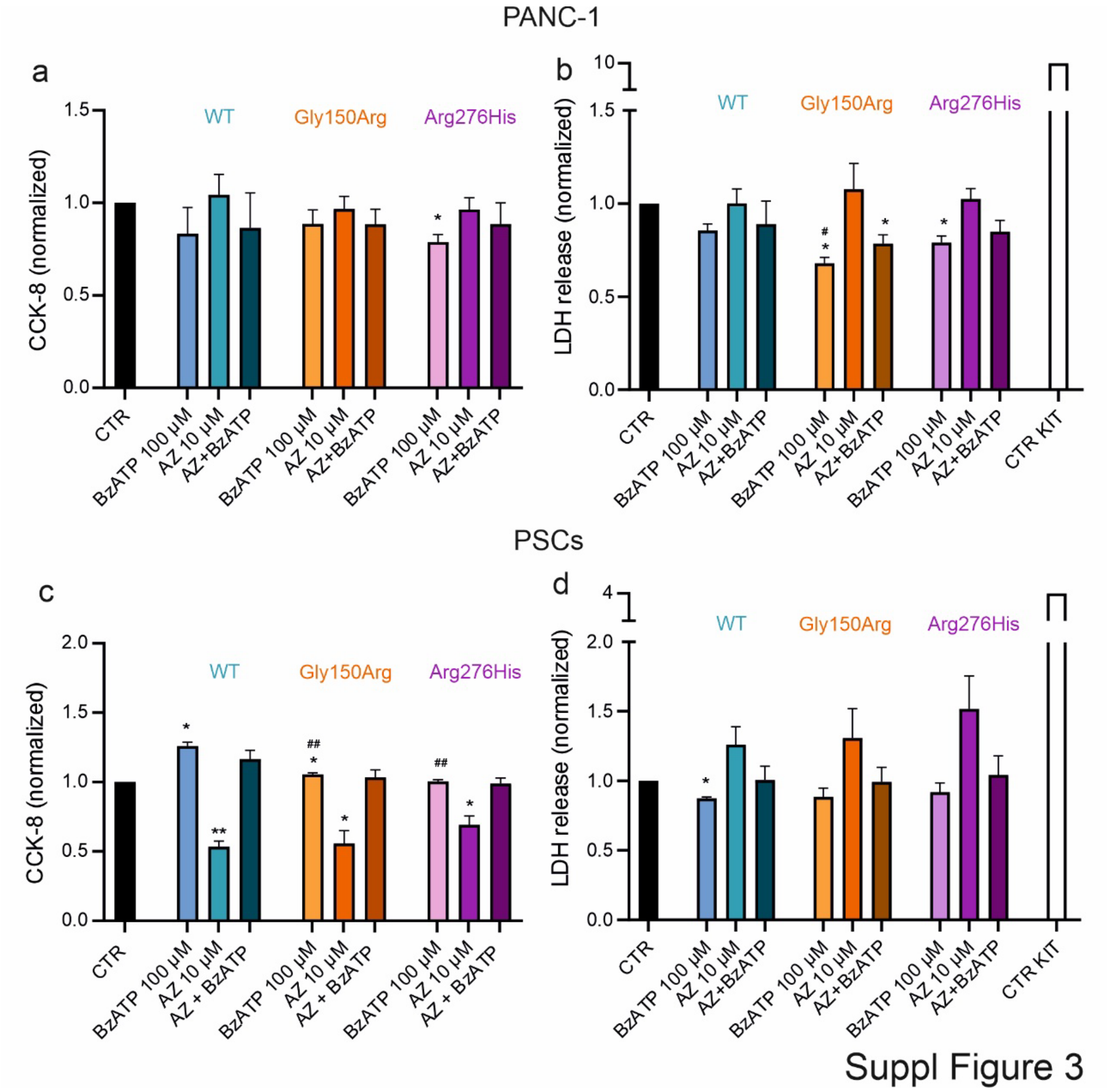

Figure S3. **Effect of P2X7R SNPs on cell survival.** Evaluation of the effect of P2X7R SNPs on cell proliferation and LDH release in (a-b) PANC-1 and (c-d) PSCs. In each graph, data shows the mean ± SEM of n=3 independent experiments. Cell proliferation was evaluated by measuring the CCK-8 incorporation after stimulation with BzATP 100 µM and pre-treatment with AZ10606120 (AZ) 10 µM with/without BzATP in **(a)** PANC-1 and **(c)** PSCs. LDH release was evaluated in the given conditions for **(b)** PANC-1 and **(d)** PSCs. The LDH release data also shows the positive control supplied with the kit (CTR KIT). All data were normalized with respect to their respective control condition (CTR) and significance was evaluated with one-sample t-test and represented as followed: *p< 0.05; **p< 0.01. Comparison of the same treatments (BzATP) between WT and the different mutants has been performed using t-test and represented as #< 0.05; ##< 0.01. Cell proliferation and cytotoxicity assays were done as follows. PANC-1 (12.000) and PSCs (30.000) were seeded in and 96-well plate and 24-well plate in complete media, respectively. After 24 h the media was discarded and substituted with fresh media containing 1% FBS and P2X7R antagonist and/or agonist and incubated at 37 °C, 5% CO2 for 48 h. At this point, the media was collected and used for the cytotoxicity assay, In Vitro Toxicology Assay Kit Lactate Dehydrogenase (LDH) based (Invitrogen, C20300), according to the manufacturer's instructions. Absorbance was measured in a FLUOstar Optima microplate reader (BMG Labtech, Ortenberg, DE). To quantify the number of cells, and thus the proliferation rate, the same cells were washed and incubated with CCK-8 (Sigma, 96992) for 1.5 h according to the manufacturer's instructions and absorbance was measured in a FLUOstar Optima microplate reader.

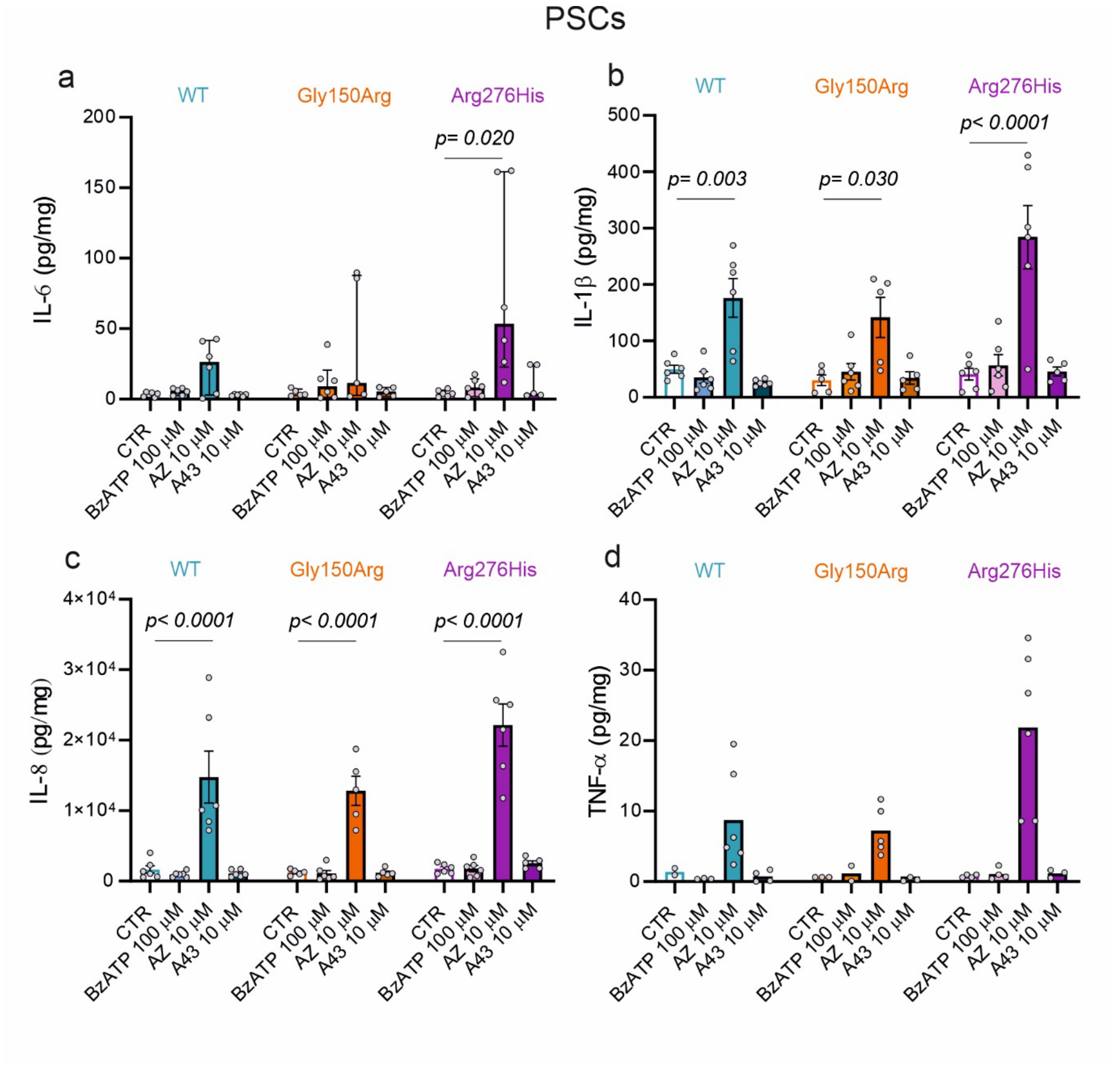

Suppl Figure 4

Figure S4. **Cytokines quantification in PSCs lysates.** (a-d) IL-6, IL-1β, IL-8 and TNF-α concentration in cell lysate of PSCs transfected with WT (blue), Gly150Arg (orange) and Arg276His (magenta) receptors. Values are reported as pg/mg after normalization on the total amount of extracted proteins. Cells were treated with BzATP 100 μM, AZ10606120 (AZ) 10 μM and A438079 (A43) 10 μM. Unstimulated cells are reported as CTR. The data are shown as the mean ± SEM for IL-1β and IL-8 and median ± interquartile range for IL-6, of n=3. Mean is also reported for TNF-α data (n=2 and 6). IL-1β and IL-8 statistics have been performed with one-way ANOVA; IL-6 statistics have been performed with Kruskal-Wallis test. The p-values are reported in the graphs.
