# Supplementary material for: Human P2X7 receptor variants Gly150Arg and Arg276His polymorphisms have differential effects on risk association and cellular functions in pancreatic cancer
Source: Cancer Cell Int. 2024 Apr 25;24:148. doi: 10.1186/s12935-024-03339-9 (PMC11044319; doi:10.1186/s12935-024-03339-9)
Supplement: Supplementary file 2 — Additional file 2: Table S1 Frequency of common nonsynonymous P2X7R-SNPs; Table S2 P2X7R-SNP analysis in pancreatic cell lines; Table S3 Ca2+ signals in P2X7R-GFP cells. [file 12935_2024_3339_MOESM2_ESM.pdf]

Supplementary Table 1 Frequency of common nonsynonymous P2X7R-SNPs

ID	Base change	AA Change	Short form	HWE value	MAF (PDAC)	MAF (Controls)
rs35933842	P2X7_151+1g>t	Null allele	P2X7l	1	0.007	0.01
rs17525809	P2X7_253T>C	Val76Ala	V76A	1	0.073	0.063
rs28360447	P2X7_474G>A	Gly150Arg	G150R	1	0.013	0.021
rs7958311	P2X7_835G>A	Arg270His	R270H	0.83	0.233	0.254
rs7958316	P2X7_853G>A	Arg276His	R276H	1	0.016	0.016
rs28360457	P2X7_946G>A	Arg307Gln	R307Q	1	0.011	0.012
rs1718119	P2X7_1068G>A	Ala348Thr	A348T	0.79	0.416	0.398
rs2230911	P2X7_1096C>G	Thr357Ser	T357S	0.85	0.08	0.086
rs2230912	P2X7_1405A>G	Gln460Arg	Q460R	0.66	0.162	0.167
rs3751143	P2X7_1513A>C	Glu496Ala	E496A	0.31	0.166	0.166
rs1653624	P2X7_1729T>A	Ile568Asn	I568N	0.97	0.027	0.034

Mutation identity, SNP notation, Hardy-Weinberg equilibrium (HWE) values and Minor Allele Frequency (MAF)

Supplementary Table 2 P2X7R-SNP analysis in pancreatic cell lines

ID	AA change	Base change	ASPC1	BxPC3	Capan-1	HPDE	Mia_Paca	PANC-1	PS1	RLT-PSCs	Function
rs35933842	Null allele	P2X7_151+1g>t	G:G	G:G	G:G	G:G	G:G	G:G	G:G	G:G	LOF
rs17525809	Val76Ala	P2X7_253T>C	T:T	**T:C**	T:T	T:T	T:T	T:T	T:T	T:T	LOF
rs28360447	Gly150Arg	P2X7_474G>A	G:G	G:G	G:G	G:G	G:G	G:G	**G:A**	G:G	LOF
rs7958311	Arg270His	P2X7_835G>A	G:G	**G:A**	G:G	**A:A**	G:G	G:G	G:G	G:G	GOF/LOF
rs7958316	Arg276His	P2X7_853G>A	G:G	G:G	G:G	G:G	G:G	G:G	G:G	G:G	LOF
rs28360457	Arg307Gln	P2X7_946G>A	G:G	G:G	G:G	G:G	G:G	G:G	G:G	G:G	LOF
rs1718119	Ala348Thr	P2X7_1068G>A	G:G	**G:A**	**A:A**	?	G:G	**A:A**	G:A	A:A	GOF
rs2230911	Thr357Ser	P2X7_1096C>G	C:C	C:C	C:C	C:C	**G:G**	C:C	C:C	C:C	LOF
rs2230912	Gln460Arg	P2X7_1405A>G	A:A	A:A	A:A	A:A	A:A	**G:G**	A:A	A:A	LOF
rs3751143	Glu496Ala	P2X7_1513A>C	A:A	A:A	A:A	A:A	A:A	A:A	A:A	A:A	LOF
rs1653624	Ile568Asn	P2X7_1729T>A	T:T	T:T	T:T	T:T	T:T	T:T	T:T	T:T	LOF
rs208294	His155Tyr	P2X7_489C>T	**T:T**	C:C	C:C	**T:C**	**T:T**	**T:T**	**T:C**	?	GOF

Polymorphisms within the P2X7 gene in 6 pancreatic cancer cells lines and 2 pancreatic stellate cell lines are indicated in bold. Two cell lines used in the study arePANC-1 and RTL-PSCs. P2X7R variant loss of lunction (LOF) and gain in of function (GOF) documented from studies of other cell type are indicated

Supplementary Table 3 Ca^{2+} signals in P2X7R-GFP cells

	PANC-1			RLT-PSCs			HEK293		
P2X7R	n. cells	Resp BzATP	Peak	n. cells	Resp BzATP	Peak	n. cells	Resp BzATP	Peak
WT	24	92%	0.57	27	78%	0.52	26	100%	0.55
Gly150Arg	23	17%	0.30	29	55%	0.15	17	53%	0.19
Arg276His	22	100%	0.53	26	77%	0.47	20	100%	0.43

Data is from 3-4 independent experiments with number of cells analyzed and showing response to BzATP (in %) and the average peak Fura-2 response
